# Gut microbiota–atrium axis: Microbiota metabolite-mediated regulation of the NLRP3 inflammasome in atrial fibrillation—pathophysiological roles and therapeutic perspectives

**DOI:** 10.1016/j.hroo.2026.05.010

**Published:** 2026-05-21

**Authors:** Xiu Ling, Jiancheng Hu

**Affiliations:** 1 Department of Cardiology, The Second Affiliated Hospital of Wannan Medical University, Wuhu, Anhui Province, China; 2 The First Affiliated Hospital of USTC, Division of Life Sciences and Medicine, University of Science and Technology of China, Hefei, Anhui, China

**Keywords:** Atrial fibrillation, NLRP3 inflammasome, Gut microbiota, Microbial metabolites, lipopolysaccharide, Short-chain fatty acid, Trimethylamine N-oxide

## Abstract

The NLR family pyrin domain containing 3 (NLRP3) inflammasome has been increasingly implicated in the pathophysiology of atrial fibrillation, whereas gut microbial metabolites contribute to systemic inflammatory signaling. This review proposes a mechanistic framework linking microbiota-derived metabolites to NLRP3 activation, with the recognition that much of the evidence derives from immune or noncardiac models rather than atrial tissue. We organize the discussion across the stages of NLRP3 activation—priming, assembly, and downstream signaling—and summarize how key metabolites may differentially modulate these processes. Lipopolysaccharide and indoxyl sulfate have been associated with enhanced inflammasome activation, whereas short-chain fatty acids and selected bile acids exhibit inhibitory or regulatory effects. Trimethylamine N-oxide has been implicated in oxidative stress–related pathways, although direct atrial evidence remains limited. Based on these insights, we present a 4-tier conceptual framework to highlight potential intervention strategies while acknowledging the predominantly preclinical nature of the current evidence.

**Key Findings**

▪ Gut microbial metabolites seem to regulate atrial fibrillation–relevant inflammation in a stage-specific manner through the NLR family pyrin domain containing 3 inflammasome.
▪ Lipopolysaccharide and indoxyl sulfate mainly support priming, whereas trimethylamine N-oxide and selected bile acids are more closely linked to inflammasome assembly.
▪ Short-chain fatty acids may counter both priming and assembly by strengthening barrier function and suppressing nuclear factor kappa-B–related signaling.
▪ Current human evidence is largely associative, and many mechanistic links remain supported mainly by preclinical or nonatrial models.
▪ A stratified therapeutic framework can help separate established lifestyle strategies from exploratory metabolite-targeting and inflammasome-directed interventions.

## Introduction

Despite advances in catheter ablation and pharmacotherapy for atrial fibrillation (AF), recurrence rates remain substantial, prompting continued investigation into mechanisms beyond traditional risk factors. The NLR family pyrin domain containing 3 (NLRP3) inflammasome has been increasingly implicated as a contributor to AF pathophysiology, potentially integrating diverse upstream signals into pathways of atrial inflammation and structural remodeling.^1^, ^2^, ^3^ Concurrently, the gut microbiota is recognized as an important regulator of systemic inflammatory processes through its diverse metabolites.^4^^,^^5^ Experimental studies have shown that several microbiota-derived metabolites can influence NLRP3 inflammasome activity in immune, endothelial, and other noncardiac cell types.^6^^,^^7^ However, whether these mechanisms operate directly in atrial tissue remains incompletely defined.

In this review, we adopt a mechanistically oriented perspective. Rather than cataloging individual microbial metabolites and their associations with AF, we organize the discussion according to the sequential stages of NLRP3 inflammasome activation. In particular, we consider how microbiota-derived metabolites may influence priming signals, inflammasome assembly, and downstream effector pathways.

We further examine potential therapeutic strategies that could target these mechanistic nodes, with an emphasis on distinguishing exploratory approaches from those supported by emerging clinical evidence. We aimed to provide a structured framework for understanding how the gut microbiota may contribute to AF pathophysiology, while highlighting areas where further mechanistic and translational validation is needed.

## The NLRP3 inflammasome: A 3-step activation process with functional consequences in the atria

Before discussing how microbial metabolites affect the NLRP3 inflammasome, we first describe the stepwise process of NLRP3 activation, which can be divided into 3 stages: priming, assembly, and downstream signaling. Importantly, we then extend this framework to cover the specific electrophysiological (EP), autonomic, and structural consequences that are critical for AF pathogenesis.

### Priming

The priming signal is typically provided by Toll-like receptor ligands, such as lipopolysaccharide (LPS), or inflammatory cytokines, such as tumor necrosis factor α. This signal activates nuclear factor kappa-B (NF-κB), leading to increased transcription of NLRP3, pro-caspase-1, and pro–interleukin-1 beta (IL-1β).^8^^,^^9^ Without this step, cells lack sufficient inflammasome components to mount a response.

### Assembly

The activation signal triggers assembly of the inflammasome complex. This signal can take many forms: extracellular adenosine triphosphate (acting through P2X7 receptors), mitochondrial reactive oxygen species (mtROS), potassium efflux, or crystalline structures that cause lysosomal damage.^10^, ^11^, ^12^ These diverse stimuli converge on NLRP3 oligomerization, recruitment of the adaptor protein apoptosis-associated speck-like protein containing a caspase recruitment domain, and activation of caspase-1.

### Downstream signaling

Activated caspase-1 cleaves pro-IL-1β and pro-IL-18 into their active forms, which are then secreted. Caspase-1 also cleaves gasdermin D (GSDMD); the N-terminal fragment forms pores in the cell membrane, leading to pyroptosis and release of proinflammatory contents.^13^^,^^14^

### EP consequences of NLRP3 downstream effectors

#### Ryanodine receptor 2 and calcium handling

IL-1β has been reported to influence calcium/calmodulin-dependent protein kinase II–related signaling pathways in experimental systems. Calcium/calmodulin-dependent protein kinase II activation has been shown to promote phosphorylation of the ryanodine receptor 2 at serine 2814, which has been associated with increased channel open probability and diastolic Ca^2+^ leak.

The resultant elevation in diastolic Ca^2+^ has been linked to delayed afterdepolarizations and triggered activity, well-established mechanisms contributing to atrial arrhythmogenesis.^15^

#### Ion channel remodeling

IL-1β has been shown to modulate ion channel function in experimental and nonatrial models. For example, downregulation of L-type calcium current through post-translational modification of Cav1.2 subunits has been reported, which may contribute to action potential shortening and increased susceptibility to reentry.

Similarly, the ultrarapid delayed rectifier potassium current, which plays a key role in atrial repolarization, has been reported to be reduced under inflammatory conditions, potentially involving transcriptional regulation of Kv1.5 channels.^16^ Collectively, these alterations have been associated with shortening of the atrial effective refractory period and increased dispersion of repolarization in experimental systems.

#### Gap junctions

GSDMD pore formation has been associated with membrane permeabilization and downstream inflammatory signaling, which may influence intercellular coupling in experimental models. Redistribution of connexin 40 and connexin 43 has been observed under inflammatory conditions, with lateralization from intercalated discs to the lateral membrane.

Such changes may disrupt anisotropic conduction and slow local conduction velocity, thereby contributing to the formation of a substrate for reentrant arrhythmias.^17^

### Autonomic nervous system and ganglionated plexi modulation

The gut–brain–heart axis involves complex autonomic regulatory pathways. Microbial metabolites such as trimethylamine N-oxide (TMAO) and LPS have been reported to influence autonomic signaling in experimental systems, including modulation of vagal afferent pathways and cardiac ganglionated plexi (GPs).

In animal models, local administration of TMAO to atrial GP has been associated with increased sympathetic activity and reduced parasympathetic tone, accompanied by shortening of the atrial effective refractory period and increased AF inducibility.^18^ Mechanistically, TMAO has been reported to activate NF-κB signaling in experimental settings, which may contribute to increased production of inflammatory mediators and enhanced GP excitability.

These findings suggest a potential link between gut-derived metabolites and neural regulation of atrial EP, although direct evidence in human atrial tissue remains limited.

### Atrial structural remodeling and fibrosis

The transition from paroxysmal to persistent AF has been associated with structural remodeling of the atria. NLRP3-derived IL-1β has been reported to activate fibroblast-related signaling pathways in experimental models and may contribute to atrial fibrosis.

Engagement of IL-1β with its receptor IL-1R1 has been associated with activation of the transforming growth factor β/Smad2/3 signaling pathway, promoting myofibroblast differentiation and extracellular matrix deposition, including collagen types I and III. In addition, IL-1β has been linked to activation of platelet-derived growth factor receptor signaling, which may further enhance fibroblast proliferation and migration.^19^

These processes have been associated with the development of a structural substrate that supports AF maintenance. This provides a mechanistic rationale for considering interventions targeting IL-1β or NLRP3 in advanced stages of AF, although clinical validation remains limited.

## Microbial metabolites that modulate NLRP3 priming

Priming determines the baseline readiness of the inflammasome. Metabolites that enhance priming increase the cellular pool of NLRP3, pro-caspase-1, and pro-IL-1β, making cells more responsive to subsequent activation signals. Metabolites that suppress priming reduce this baseline readiness.

### LPS: The prototypical priming signal

LPS, a component of gram-negative bacteria, has been widely used as a priming stimulus for NLRP3 inflammasome activation, particularly in immune cell models. It activates Toll-like receptor 4 (TLR4) signaling and downstream NF-κB pathways, leading to upregulation of inflammasome components.^20^^,^^21^ Although TLR4 expression has been reported in cardiac tissues, including atrial cells, much of the mechanistic evidence for LPS-induced NLRP3 priming derives from immune or noncardiac systems.

In clinical studies, circulating LPS levels have been reported to be elevated in patients with AF and are associated with adverse outcomes.^22^ However, these observations remain correlative and do not establish a direct causal role for LPS in atrial remodeling.

Increased circulating LPS levels are thought to be linked to impaired intestinal barrier function. Conditions such as a high-fat diet, obesity, and aging have been associated with increased intestinal permeability, which may facilitate the translocation of LPS into the circulation.^23^^,^^24^ In a microbiota transfer model, Zhang et al^22^ demonstrated that transplantation of microbiota from aged to young rats was associated with increased circulating LPS levels, enhanced atrial NLRP3 expression, and greater susceptibility to AF; these effects were attenuated by the NLRP3 inhibitor MCC950. These findings support a potential link between gut-derived endotoxemia and atrial inflammasome signaling in experimental settings, although their direct relevance to human AF remains to be established.

### Short-chain fatty acids: Suppressors of priming

Short-chain fatty acids (SCFAs)—acetate, propionate, and butyrate—are produced from dietary fiber fermentation. They suppress NLRP3 priming by strengthening the intestinal barrier (reducing LPS translocation) and directly inhibiting NF-κB via histone deacetylase inhibition.^24^, ^25^, ^26^, ^27^ In patients with AF, fecal SCFA levels are lower than in controls and inversely correlate with AF burden.^27^ In animal models, SCFA supplementation reduced AF inducibility and atrial NLRP3 expression.^27^ Dietary fiber intake also enriches beneficial gut bacteria.^28^

### Indoxyl Sulfate: A priming signal in chronic kidney disease

Indoxyl sulfate (IS), a uremic toxin derived from tryptophan metabolism, accumulates in chronic kidney disease (CKD) and has been implicated in cardiovascular dysfunction. Experimental studies have shown that IS can activate NF-κB signaling and upregulate NLRP3 inflammasome components in cardiac and other cell types, although much of this evidence derives from in vitro or nonatrial models.^29^^,^^30^ Patients with CKD have a markedly increased risk of AF, and elevated IS levels have been proposed as 1 of several contributing factors linking renal dysfunction to cardiovascular remodeling.^30^^,^^31^ In addition to overt CKD, IS accumulation may also occur in conditions associated with impaired renal clearance or metabolic dysfunction, which are frequently present in AF populations.

Although these observations suggest a potential role for IS in promoting proinflammatory priming pathways, the extent to which IS directly contributes to atrial NLRP3 inflammasome activation and AF pathogenesis remains incompletely defined.

## Microbial metabolites that modulate NLRP3 assembly

After priming, a second signal triggers inflammasome assembly. This is the critical decision point: a primed cell that does not receive an activation signal will not undergo inflammasome activation. Microbial metabolites can either promote or inhibit assembly.

### TMAO: A promoter of inflammasome assembly

TMAO is generated from dietary choline and L-carnitine through gut microbiota–dependent metabolism, in which these precursors are converted to trimethylamine and subsequently oxidized in the liver.^32^^,^^33^ Elevated circulating TMAO levels have been associated with increased cardiovascular risk in multiple clinical studies.^34^^,^^35^

Experimental studies suggest that TMAO may influence NLRP3 inflammasome activation through pathways involving mitochondrial oxidative stress. In particular, TMAO has been reported to promote mtROS production via the SIRT3–SOD2 axis in endothelial and other noncardiac cell models, although direct evidence in atrial tissue remains limited.^36^^,^^37^

In animal models, TMAO has been linked to EP and inflammatory changes relevant to AF. For example, local administration of TMAO to atrial GPs in canine models has been associated with EP instability and increased AF susceptibility.^18^ In a rat model, cold exposure was accompanied by increased circulating TMAO levels and enhanced atrial caspase-1 activation, whereas genetic deletion of caspase-1 attenuated AF susceptibility under these conditions.^38^

Together, these findings suggest a potential role for TMAO in modulating inflammasome-related pathways relevant to AF; however, the underlying mechanisms remain incompletely defined, and direct causal relationships in human atrial tissue have yet to be established.

### Bile acids: Context-dependent modulators of assembly

Bile acids (BAs) are synthesized in the liver and further modified by gut microbiota into a diverse range of secondary BAs. Their potential effects on NLRP3 inflammasome activation seem to vary depending on the specific BA species and the receptors involved.

Experimental studies have suggested that chenodeoxycholic acid may promote inflammasome assembly through mechanisms involving Ca^2+^ influx, although these findings are primarily derived from noncardiac cellular models.^39^ In contrast, activation of the farnesoid X receptor by certain BAs has been reported to exert inhibitory effects on inflammasome activation pathways.^39^

In clinical settings, patients with AF have been reported to exhibit altered BA profiles. Elevated circulating chenodeoxycholic acid levels have been associated with left atrial remodeling, whereas ursodeoxycholic acid levels are reduced.^40^^,^^41^ However, these observations are correlative and do not establish a causal relationship between specific BAs and atrial structural changes.

Whether BA-mediated effects on atrial remodeling are directly linked to NLRP3 inflammasome activation remains to be determined.

### SCFAs: Inhibitors of assembly

SCFAs also inhibit the assembly step. Zuo et al^27^ showed that SCFA supplementation reduced AF inducibility in a pacing-induced model, an effect dependent on the SCFA receptor GPR43. Thus, SCFAs uniquely inhibit both priming and assembly.

## Microbial metabolites that modulate downstream effectors

### LPS and the noncanonical pathway

In addition to its role in priming through TLR4 signaling, LPS has been shown in experimental models to activate noncanonical inflammasome pathways after cytosolic entry. In this context, intracellular LPS can directly activate caspase-11 in mice (or caspase-4/5 in humans), leading to cleavage of GSDMD and induction of pyroptotic membrane permeabilization.^13^^,^^14^

This process has been associated with potassium efflux and secondary activation of the NLRP3 inflammasome, thereby amplifying inflammatory signaling. Although this pathway is well characterized in immune cells, its relevance to cardiac tissues, including atrial cardiomyocytes, remains incompletely defined. As such, its contribution to AF should be considered hypothesis generating rather than established.

### GSDMD as a direct target

Emerging evidence suggests that GSDMD may exert functions beyond its established role in pyroptosis. In a recent experimental study, Yuan et al^42^ reported that the N-terminal GSDMD fragment can localize to mitochondria in atrial cardiomyocytes, where it was associated with increased mtROS production and disrupted calcium handling.

These findings suggest a potential role for GSDMD in modulating EP properties independent of cell membrane pore formation. However, these observations are currently limited to specific experimental settings, and further studies are needed to determine their generalizability and relevance to human AF. Consequently, the impact of microbial metabolites on GSDMD-mediated pathways remains an area for further investigation.

## A stratified therapeutic framework targeting the gut–NLRP3–AF axis

**Figure 1:**
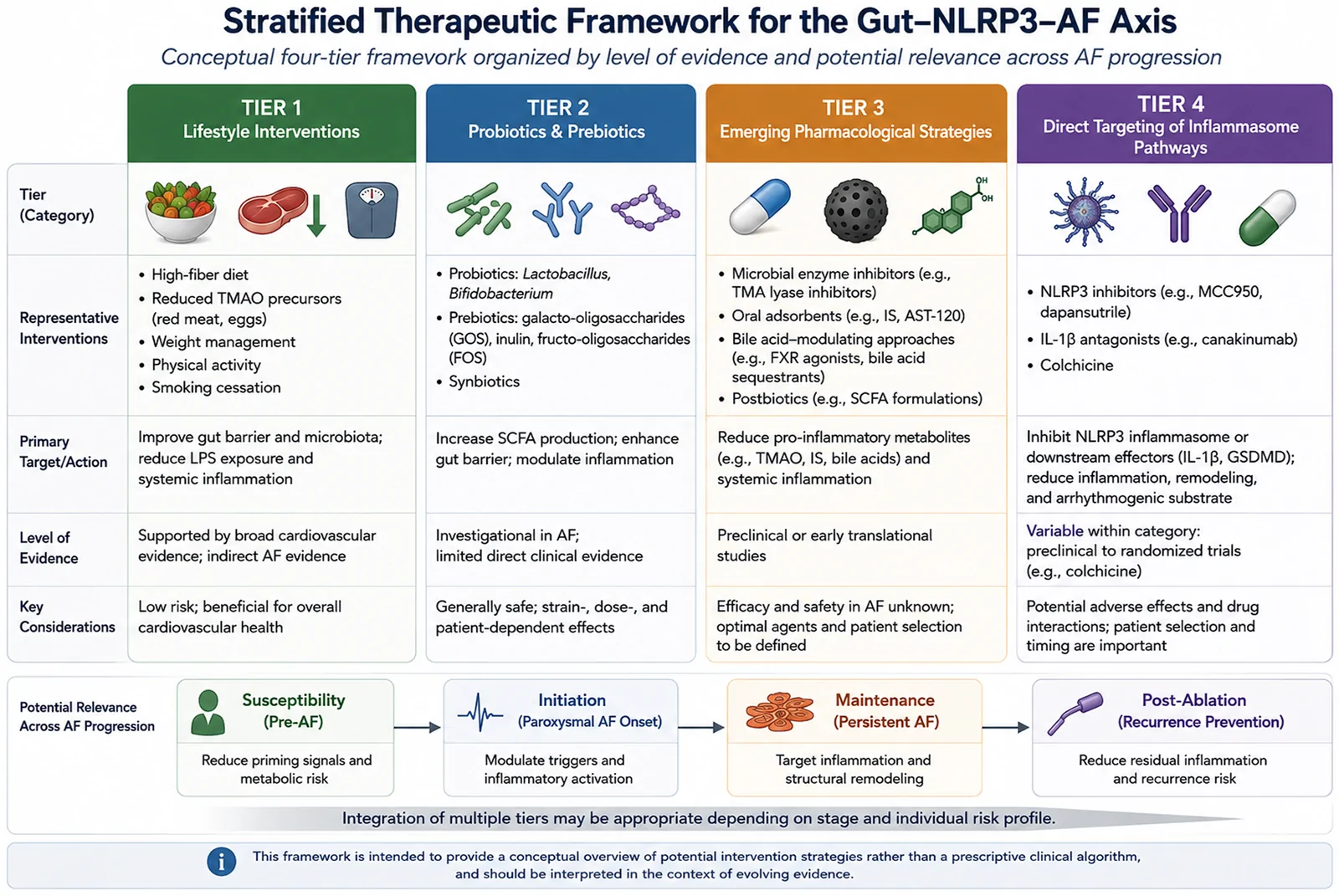
Stratified therapeutic framework targeting the gut–NLRP3–AF axis. Conceptual 4-tier framework for interventions targeting the gut–NLRP3–AF axis, organized according to approximate level of clinical evidence and potential relevance across stages of AF progression. Tier 1 (lifestyle interventions) includes high-fiber dietary patterns, reduction of TMAO precursor intake, and weight management. These approaches are supported by general cardiovascular evidence and may influence upstream inflammatory pathways. Tier 2 (probiotics and prebiotics) includes strains such as Lactobacillus and Bifidobacterium, as well as substrates such as galacto-oligosaccharides. These interventions are considered investigational in the context of AF, with limited direct clinical evidence. Tier 3 (emerging pharmacologic strategies) includes microbial enzyme inhibitors, oral adsorbents, and bile acid–modulating approaches, which are primarily supported by preclinical or early-stage translational studies. Tier 4 (direct targeting of inflammasome pathways) includes specific NLRP3 inhibitors (eg, MCC950, dapansutrile), IL-1β antagonists (eg, canakinumab), and colchicine. The level of clinical evidence varies substantially within this category, ranging from preclinical studies to randomized clinical trials, and safety considerations remain important. The framework is intended to provide a conceptual overview of potential intervention strategies rather than a prescriptive clinical algorithm and should be interpreted in the context of evolving evidence. AF = atrial fibrillation; IL-1β = interleukin-1 beta; NLRP3 = NLR family pyrin domain containing 3; TMAO = trimethylamine N-oxide.

The stepwise nature of NLRP3 inflammasome activation—priming, assembly, and downstream signaling—provides a useful framework for considering potential points of intervention. However, available strategies differ substantially in their level of clinical maturity and supporting evidence. A 4-tier conceptual framework that organizes these approaches according to their translational readiness and evidentiary support, ranging from lifestyle modifications (tier 1) to direct targeting of inflammasome components (tier 4), is presented in Figure 1.

### Tier 1: Lifestyle interventions

Lifestyle-based strategies represent the most established and broadly applicable approaches, with well-documented benefits for cardiovascular health. Although their specific effects on AF through NLRP3-related pathways remain incompletely defined, several interventions may indirectly influence inflammasome activation.

A high-fiber diet has been associated with increased production of SCFAs, which have been reported to inhibit both priming and assembly of the NLRP3 inflammasome in experimental models.^24^, ^25^, ^26^, ^27^ In addition, high-fiber intake may help maintain intestinal barrier integrity, potentially reducing systemic exposure to LPS.^26^^,^^43^ Dietary patterns such as the Mediterranean diet provide a practical framework for achieving these changes and have also been associated with modulation of gut microbiota composition.^44^ Reduced intake of red meat and other dietary sources of trimethylamine precursors has been associated with lower circulating TMAO levels, although direct evidence linking these changes to AF outcomes remains limited.^32^^,^^33^ Weight reduction is another clinically supported intervention, given that obesity has been associated with increased intestinal permeability and AF burden in clinical studies.^23^^,^^24^^,^^45^

### Tier 2: Probiotics and prebiotics

Probiotics and prebiotics have been proposed as strategies to modulate gut microbiota composition and function. Experimental evidence suggests that bacterial strains such as Lactobacillus and Bifidobacterium may enhance intestinal barrier integrity and reduce circulating endotoxin levels.^46^ Prebiotic compounds, including galacto-oligosaccharides, have been associated with increased SCFA production.^47^ However, clinical evidence supporting their efficacy in reducing AF incidence or recurrence remains limited, and optimal strain selection, dosing, and target populations have not been clearly defined.

### Tier 3: Targeting microbial metabolites

Strategies aimed at directly modulating microbiota-derived metabolites represent an emerging area of investigation. 1 approach involves inhibition of microbial enzymes responsible for the production of trimethylamine, a precursor of TMAO. Experimental studies have demonstrated that such inhibition can reduce circulating TMAO levels and attenuate atherosclerosis in animal models.^48^ Whether these interventions influence AF-related outcomes remains unknown.

Additional approaches include the use of oral adsorbents to reduce circulating levels of uremic toxins such as IS, particularly in the context of renal dysfunction.^49^ Although such interventions have been associated with reductions in oxidative stress, their effects on AF susceptibility have not been established. Modulation of BA composition has also been proposed; however, available data are heterogeneous, with different BA species exerting divergent effects on inflammasome activation pathways.^39^, ^40^, ^41^

### Tier 4: Direct targeting of inflammasome pathways

Direct modulation of the NLRP3 inflammasome or its downstream mediators represents a more mechanistically specific approach, although these strategies remain at varying stages of development.

Small-molecule NLRP3 inhibitors, such as MCC950, have been shown to inhibit inflammasome activation and reduce AF susceptibility in experimental models.^50^^,^^51^ Dapansutrile (OLT1177) has advanced to early-phase clinical trials and has demonstrated a favorable safety profile in patients with cardiovascular conditions, although its efficacy in AF remains to be established.^52^^,^^53^

Targeting downstream inflammatory mediators has also been explored. IL-1β inhibition with canakinumab has demonstrated cardiovascular benefit in large-scale clinical trials, although AF-specific data remain limited and an increased risk of infection has been reported.^54^^,^^55^

Colchicine, a widely available anti-inflammatory agent with inhibitory effects on inflammasome activation, has been evaluated in clinical studies of AF with mixed results.^56^^,^^57^ Although some studies suggest a reduction in postoperative AF, others have not demonstrated consistent benefit, and tolerability may limit its use in certain patients.

Overall, although inflammasome-targeted strategies show mechanistic promise, their clinical applicability in AF requires further validation through adequately powered and disease-specific clinical studies.

## Integrating dynamics and stratification: A conceptual framework

**Figure 2:**
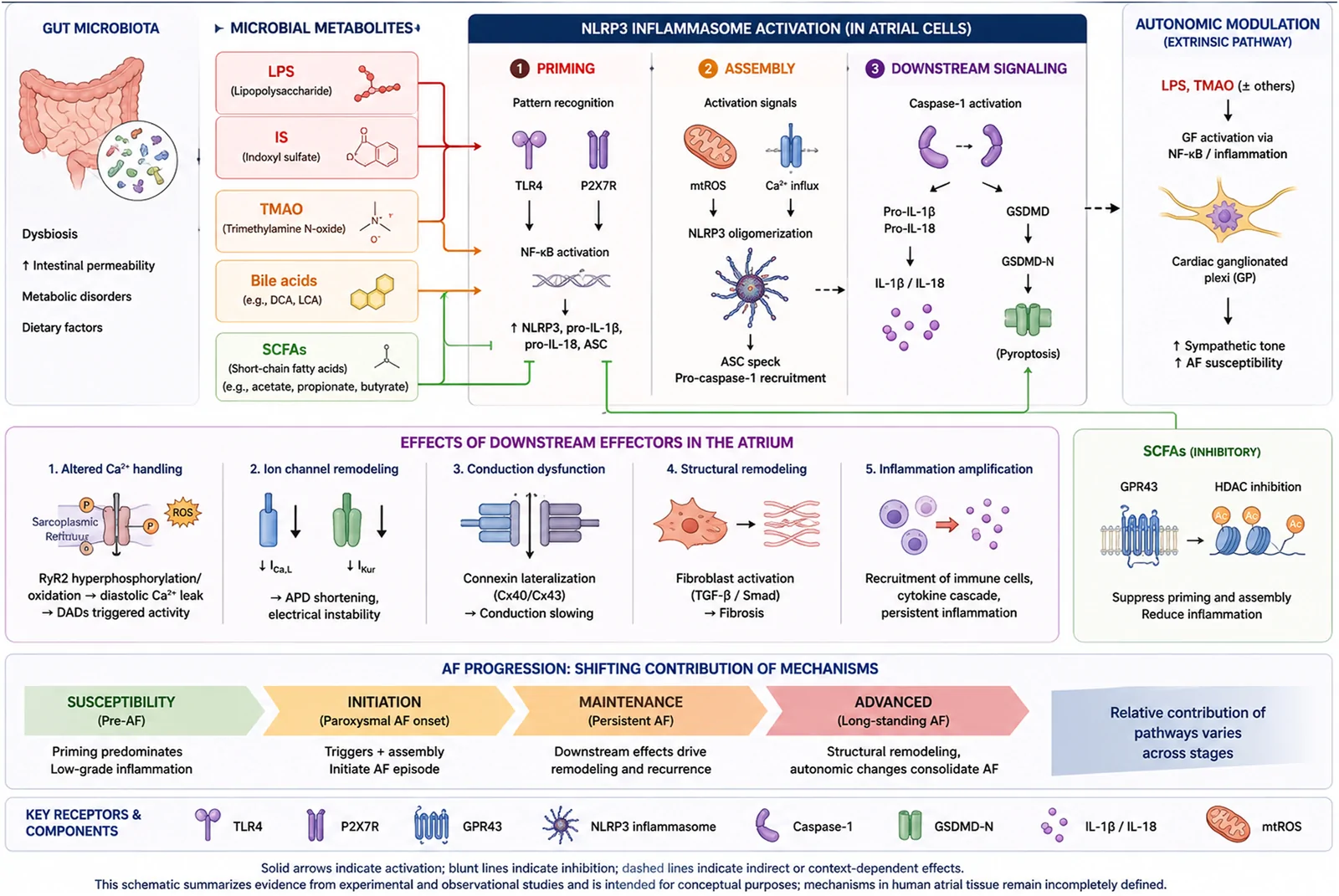
Mechanistic framework of the gut–NLRP3–AF axis across AF progression. Schematic illustration of the proposed interactions between gut microbial metabolites and NLRP3 inflammasome signaling across different stages of AF. The diagram is organized around the 3 stages of inflammasome activation—priming, assembly, and downstream signaling—and summarizes how selected metabolites have been reported to modulate these processes in experimental systems. LPS, acting via TLR4, and IS have been associated with priming of inflammatory signaling pathways. TMAO and certain bile acids have been reported to influence inflammasome assembly, potentially through mechanisms involving mtROS and calcium signaling. In contrast, SCFAs have been shown to exert inhibitory effects on inflammatory signaling, including pathways involving GPR43 and HDAC inhibition. Downstream effectors such as IL-1β and GSDMD have been linked in experimental models to multiple processes relevant to atrial arrhythmogenesis, including altered calcium handling, ion channel remodeling, gap junction redistribution, and fibroblast activation. These processes have been associated with electrophysiological instability and structural remodeling; however, the specific molecular pathways and their relative contributions in human atrial tissue remain incompletely defined. In parallel, microbial metabolites such as TMAO and LPS have been reported to modulate autonomic signaling, including effects on cardiac GPs, potentially through inflammatory pathways. The relative contribution of these mechanisms may vary across stages of AF progression, from susceptibility and initiation to maintenance. Key receptors and signaling components (eg, TLR4, P2X7, GPR43) are *highlighted* for illustrative purposes. This schematic is intended as a conceptual summary of current evidence rather than a definitive representation of established mechanisms. AF = atrial fibrillation; GP = ganglionated plexus; GSDMD = gasdermin D; HDAC = histone deacetylase; IL-1β = interleukin-1 beta; IS = indoxyl sulfate; LPS = lipopolysaccharide; mtROS = mitochondrial reactive oxygen species; NLRP3 = NLR family pyrin domain containing 3; SCFA = short-chain fatty acid; TLR4 = Toll-like receptor 4; TMAO = trimethylamine N-oxide.

Integrating the stepwise activation of the NLRP3 inflammasome with the clinical progression of AF provides a conceptual framework for considering stage-specific mechanisms and potential intervention points. Although this alignment is not definitive and remains largely hypothesis generating, it may help contextualize how distinct biological processes contribute across different phases of AF. How gut-derived metabolites—such as LPS, SCFAs, TMAO, and BAs—are reported to modulate priming, assembly, and downstream signaling pathways and how these processes may relate to atrial EP instability, structural remodeling, and autonomic modulation across different stages of AF are schematically presented in Figure 2.

### Early stage: Susceptibility and initiation

In individuals with metabolic risk factors such as obesity, dietary imbalance, or metabolic syndrome, increased intestinal permeability and altered microbial metabolite profiles have been reported. Elevated circulating LPS levels may contribute to priming of inflammatory pathways, whereas reduced SCFA production may limit anti-inflammatory signaling. In this context, the atrial substrate may be more susceptible to additional triggers, although direct causal relationships remain to be established.

Interventions categorized within tier 1, including dietary modification and weight management, have been associated with improvements in cardiovascular risk profiles and may influence upstream inflammatory signaling. However, their specific effects on NLRP3-mediated pathways in atrial tissue remain incompletely defined.

### Acute triggers: Initiation of an episode

Superimposed acute stressors—such as intermittent hypoxia, hemodynamic fluctuations, or dietary changes—may interact with a primed inflammatory environment. Experimental evidence suggests that metabolites such as TMAO and BAs can modulate inflammasome-related signaling pathways, potentially influencing activation processes. However, the extent to which these mechanisms directly initiate AF episodes in humans remains uncertain.

Strategies targeting microbial metabolite production (tier 3) have been proposed in preclinical studies, although their clinical applicability for acute AF prevention has not been established.

### Established AF: Maintenance and progression

In persistent AF, structural remodeling and sustained inflammatory signaling are more prominent. Downstream effectors, including IL-1β and GSDMD, have been implicated in fibroblast activation and extracellular matrix deposition in experimental models, contributing to the development of a proarrhythmic substrate.

Interventions targeting inflammasome components or downstream cytokines (tier 4) have been evaluated in varying stages of clinical development. For example, colchicine has been assessed in randomized trials with mixed results, whereas IL-1β antagonists and direct NLRP3 inhibitors remain under investigation with more limited clinical data. These differences highlight the heterogeneity in evidence supporting individual tier 4 strategies.

### After ablation: Preventing recurrence

After catheter ablation, residual atrial remodeling and inflammatory activity may persist. Observational and experimental studies suggest that continued modulation of inflammatory and metabolic pathways could influence recurrence risk, although definitive evidence is lacking.

Combination approaches incorporating lower-risk strategies (eg, lifestyle interventions and microbiota modulation) alongside selected anti-inflammatory therapies have been proposed, but require further validation in controlled clinical studies.

Overall, this framework emphasizes that AF represents a heterogeneous condition with dynamic pathophysiological processes. The gut microbiota–NLRP3 axis provides a potential organizing concept, but its clinical translation requires careful consideration of disease stage, patient heterogeneity, and the current limitations of available evidence.

The proposed effects of individual microbial metabolites on distinct NLRP3 inflammasome stages—priming, assembly, and downstream signaling—are heterogeneous. To provide a concise overview, key gut-derived metabolites, their reported molecular mechanisms, associated atrial EP and structural consequences, corresponding therapeutic tiers, and the current level of supporting evidence are presented in Table 1.

**Table 1:** Summary of gut-derived metabolites, NLRP3 modulation, and resultant atrial pathophysiology

Metabolite	NLRP3 stage	Key molecular mechanism	Atrial EP and structural impact	Therapeutic tier	Level of evidence	Key references
LPS	Priming	Activation of TLR4/NF-κB signaling (primarily in immune/noncardiac models)	Associated with increased AF susceptibility and conduction abnormalities in experimental and clinical studies	Tier 1 (diet/weight loss)	Observational + preclinical	20, 21, 22
SCFAs	Inhibition of priming and assembly	Activation of GPR43/GPR109A signaling; HDAC inhibition (experimental models)	Associated with reduced fibrosis and improved electrophysiological stability in preclinical studies	Tier 1 (fiber) / tier 2 (prebiotics)	Preclinical + limited clinical	24, 25, 26, 27, 28
TMAO	Assembly	Reported to promote mtROS-related pathways via SIRT3–SOD2 axis (primarily in endothelial models)	Associated with proarrhythmic calcium handling and autonomic modulation in experimental models	Tier 1 (diet) / tier 3 (microbial enzyme inhibitors)	Preclinical + observational	32, 33, 34, 35, 36, 37, 38
Indoxyl sulfate	Priming	Activation of NF-κB signaling and upregulation of inflammasome components (in vitro and nonatrial models)	Associated with atrial remodeling and fibrosis, particularly in CKD-related contexts	Tier 3 (oral adsorbents)	Preclinical + observational	29, 30, 31
BAs	Assembly	Modulation of Ca^2+^ signaling and FXR pathways (model-dependent effects)	Associated with atrial remodeling and electrophysiological alterations; effects vary by BA species	Tier 3 (BA modulators)	Preclinical + observational	39, 40, 41
Integrative downstream effectors	Downstream signaling	Downstream signaling | IL-1β / caspase-1 / GSDMD pathways	Associated with ion channel remodeling, connexin redistribution, and triggered activity in experimental systems	Tier 4 (NLRP3 inhibitors, IL-1β blockade, colchicine)	Mixed (preclinical to clinical)	13, 14, 15, 16, 17,19,42

BA = bile acid; CKD = chronic kidney disease; EP = electrophysiology; FXR = farnesoid X receptor; GP = ganglionated plexus; GSDMD = gasdermin D; HDAC = histone deacetylase; IL-1β = interleukin-1 beta; LPS = lipopolysaccharide; mtROS = mitochondrial reactive oxygen species; NF-κB = nuclear factor kappa-B; NLRP3 = NLR family pyrin domain containing 3; SCFA = short-chain fatty acid; TLR4 = Toll-like receptor 4; TMAO = trimethylamine N-oxide.

## Comparison with previous reviews

Several recent reviews have examined the relationship between gut microbiota and AF, primarily focusing on compositional changes or cataloging individual metabolites.^6^^,^^7^^,^^19^^,^^58^ Our review differs in 3 key aspects. First, we organized our discussion around the mechanistic steps of NLRP3 activation—priming, assembly, and downstream signaling—rather than individual metabolites. This framework explains how structurally unrelated metabolites converge on the same inflammatory pathway. Second, we provide an in-depth analysis of the EP, autonomic, and structural consequences of NLRP3 activation, which is critical for an EP audience. Third, we propose a stratified therapeutic framework that matches interventions to both the stage of AF progression and the level of clinical evidence, providing a clinically actionable roadmap that extends beyond descriptive associations.

## Challenges and future directions

Although the framework is supported by substantial preclinical evidence, several challenges remain.

### Causality in humans

Most human studies are cross-sectional. Prospective studies are needed to determine whether elevated proinflammatory metabolites precede AF onset and whether reducing them lowers AF risk.

### Cell-type specificity

Mechanistic studies have largely used immune cells; we need to understand how these metabolites affect atrial cardiomyocytes and fibroblasts specifically.

### Spatial considerations

The gut microbiota is not uniform along the gastrointestinal tract, and the left and right atria may respond differently to inflammatory signals.

### Personalization

Gut microbial composition varies widely. Future strategies will need to consider an individual’s baseline microbial profile and inflammatory status.

### Safety

Although tiers 1 and 2 are generally safe, tiers 3 and 4 require rigorous safety evaluation, as illustrated by the increased infection risk with canakinumab.^55^

## Conclusion

The relationship between gut microbial metabolites and NLRP3 inflammasome activation in AF can be conceptualized as involving sequential processes, including priming, assembly, and downstream signaling. Available evidence suggests that metabolites such as LPS and IS may contribute to priming, whereas TMAO and certain BAs have been associated with modulation of inflammasome assembly. In contrast, SCFAs have been reported to exert inhibitory effects on inflammatory signaling pathways.

However, much of this evidence is derived from experimental systems or nonatrial cell types, and the extent to which these mechanisms operate directly in human atrial tissue remains incompletely defined. Accordingly, the balance between proinflammatory and anti-inflammatory metabolites should be considered a conceptual framework rather than a quantitatively established determinant of AF risk.

This mechanistic perspective provides a basis for considering stratified intervention strategies. Lifestyle-based approaches and microbiota-directed interventions (tiers 1 and 2) are supported by broader cardiovascular evidence, although their specific effects on NLRP3-related pathways in AF require further clarification. Approaches targeting microbial metabolite production (tier 3) and direct inflammasome inhibition or downstream cytokines (tier 4) remain areas of active investigation, with varying levels of clinical evidence and safety considerations.

Overall, integrating gut microbiota–derived signaling with inflammasome biology offers a useful framework for understanding potential links among metabolism, inflammation, and atrial remodeling. Future studies are needed to establish causal relationships, validate these mechanisms in human atrial tissue, and determine whether targeting these pathways can meaningfully influence AF prevention or management.

## Declaration of generative AI and AI-assisted technologies in the writing process

During the preparation of this work, the authors used a large language model to assist with language refinement and expression optimization. After using this tool, the authors reviewed and edited the content as needed and take full responsibility for the content of the published article.

## Funding Sources

This research did not receive any specific grant from funding agencies in the public, commercial, or not-for-profit sectors.

## Disclosures

The authors have no conflicts of interest to disclose.
